# Light-Dependent Aerobic Methane Oxidation Reduces Methane Emissions from Seasonally Stratified Lakes

**DOI:** 10.1371/journal.pone.0132574

**Published:** 2015-07-20

**Authors:** Kirsten Oswald, Jana Milucka, Andreas Brand, Sten Littmann, Bernhard Wehrli, Marcel M. M. Kuypers, Carsten J. Schubert

**Affiliations:** 1 Department of Surface Waters—Research and Management, Eawag, Swiss Federal Institute of Aquatic Science and Technology, Kastanienbaum, Switzerland; 2 Institute of Biogeochemistry and Pollutant Dynamics, ETH Zurich, Swiss Federal Institute of Technology, Zurich, Switzerland; 3 Department of Biogeochemistry, Max Planck Institute for Marine Microbiology, Bremen, Germany; Laval University, CANADA

## Abstract

Lakes are a natural source of methane to the atmosphere and contribute significantly to total emissions compared to the oceans. Controls on methane emissions from lake surfaces, particularly biotic processes within anoxic hypolimnia, are only partially understood. Here we investigated biological methane oxidation in the water column of the seasonally stratified Lake Rotsee. A zone of methane oxidation extending from the oxic/anoxic interface into anoxic waters was identified by chemical profiling of oxygen, methane and δ^13^C of methane. Incubation experiments with ^13^C-methane yielded highest oxidation rates within the oxycline, and comparable rates were measured in anoxic waters. Despite predominantly anoxic conditions within the zone of methane oxidation, known groups of anaerobic methanotrophic archaea were conspicuously absent. Instead, aerobic gammaproteobacterial methanotrophs were identified as the active methane oxidizers. In addition, continuous oxidation and maximum rates always occurred under light conditions. These findings, along with the detection of chlorophyll *a*, suggest that aerobic methane oxidation is tightly coupled to light-dependent photosynthetic oxygen production both at the oxycline and in the anoxic bottom layer. It is likely that this interaction between oxygenic phototrophs and aerobic methanotrophs represents a widespread mechanism by which methane is oxidized in lake water, thus diminishing its release into the atmosphere.

## Introduction

Methane (CH_4_), a potent greenhouse gas with a warming potential about 20 times higher than carbon dioxide (CO_2_), is released from anthropogenic as well as natural sources with total emissions approximating 600 Tg CH_4_ a^-1^ [1]. Freshwater lakes only occupy 2–3% of Earth’s land surface [2], but they contribute substantially to atmospheric CH_4_ emissions, releasing 8–48 Tg CH_4_ a^-1^ [3]. Contrarily, oceans cover a vastly larger surface area but they only account for ~3% of global CH_4_ emissions [4]. This is not only due to the high sulfate (SO_4_^2-^) content of marine waters (28 mM), which favors SO_4_^2-^-dependent over methanogenic organic matter degradation [5], but also due to efficient aerobic and anaerobic oxidation of methane (AOM) in sediments and within the water column [6,7]. Considerably lower SO_4_^2-^ concentrations (μM range) in freshwater lakes lead to CH_4_ build-up due to methanogenesis. There is extensive evidence that methane emissions from lakes are mitigated by its oxidation, but many uncertainties about pathways and involved microorganisms, especially when oxygen is depleted, remain unanswered [8].

Microbial methane oxidation at neutral pH can be performed under both oxic and anoxic conditions. Known neutrophilic aerobic methane-oxidizing bacteria (MOB) belong to the groups of alpha- and gammaproteobacteria. Gamma-MOB (including type I and type X MOB) and alpha-MOB (including type II MOB) exhibit some metabolic differences with respect to e.g. carbon assimilation pathways [9]. To date all described MOB utilize molecular oxygen (O_2_) as the terminal electron acceptor and employ soluble- (*mmoX*) and/or particulate methane monooxygenase (*pmoA*) for methane activation and oxidation. CH_4_ oxidation linked to denitrification under apparently anoxic conditions was demonstrated in freshwater enrichment cultures [10,11]. However, the dominant bacterium from this culture (Candidatus *Methylomirabilis oxyfera*) was found to encode for the enzymatic pathway of aerobic CH_4_ oxidation and is unable to perform complete denitrification, thus suggesting that it produces O_2_ within the cell by splitting NO_x_ to N_2_ and O_2_ [12].

Microorganisms capable of directly oxidizing CH_4_ with nitrate (NO_3_^-^) as their terminal electron acceptor have also been enriched recently [13]. This candidate species, Candidatus *Methanoperedens nitroreducens*, belongs to a novel clade of known anaerobic methanotrophic archaea (ANME). Currently there are three known groups of ANME (ANME-1, -2 and -3) and even though it has been suggested that this metabolism represents a greater CH_4_ sink than the aerobic process [7], it mainly proceeds in marine environments. There, ANME occur in a consortium with various deltaproteobacteria together mediating sulfate-dependent methane oxidation (for review see Knittel and Boetius [7]). Geochemical evidence for AOM coupled to iron [Fe(III)] or manganese [Mn(IV)] reduction has been reported for marine sediments [14,15,16,17], however the mechanisms and involved microorganisms remain unresolved.

Despite the huge potential for methane turnover via AOM in marine settings, most methane oxidation in lacustrine sediments as well as in the water column has been attributed to proceed via the aerobic pathway [9,18]. In fully mixed lakes, most of the CH_4_ produced in the sediment is largely consumed at the oxygenated sediment/water interface and water column [9]. In stratified lakes exhibiting permanently (meromictic) or seasonally (mono- or dimictic) anoxic bottom waters, maximum oxidation rates and highest abundances of MOB correspond to the location of the oxic/anoxic boundary [19], indicating aerobic oxidation. Nevertheless, evidence for MO in the absence of O_2_ exists for lake environments [20,21]. It has been proposed that denitrification- [22] or iron-mediated AOM [23,24,25] could be relevant in lake sediments. Whilst bacteria related to Candidatus *Methylomirabilis oxyfera* appear to be involved in MO linked to denitrification [26], microbiological/molecular confirmation is still lacking for the iron pathway. Alternatively, in some anoxic water columns, downwelling or lateral intrusions of oxygenated water could support aerobic methane oxidation in apparently anoxic habitats [27]. Recently it was shown that methane oxidation coupled to oxygenic photosynthesis in anoxic waters is a very efficient methane filter in meromictic lakes [28]. However, the significance of this mechanism for global methane emissions is still unclear. Permanently stratified lakes are ideal for studying anoxic processes, but they are rare. In contrast seasonally stratified lakes are common world-wide, but direct evidence for MO associated with photosynthesis in these systems is still lacking.

To gain more insight into MO in freshwater systems, particularly under low light, suboxic and anoxic conditions, we investigated MO dynamics in Lake Rotsee, Central Switzerland. Lake Rotsee is a typical example of a seasonally stratified lake with respect to its size (< 1 km^2^) [2] and mixing regime [29]. We applied geochemical as well as microbiological/molecular approaches to investigate factors that influence biological methane oxidation in its hypolimnion.

## Materials and Methods

### Ethics statement

Permission for the sampling campaigns on Lake Rotsee was obtained from the Rotsee Commission, City of Lucerne, Switzerland.

### Study site

**Fig 1:**
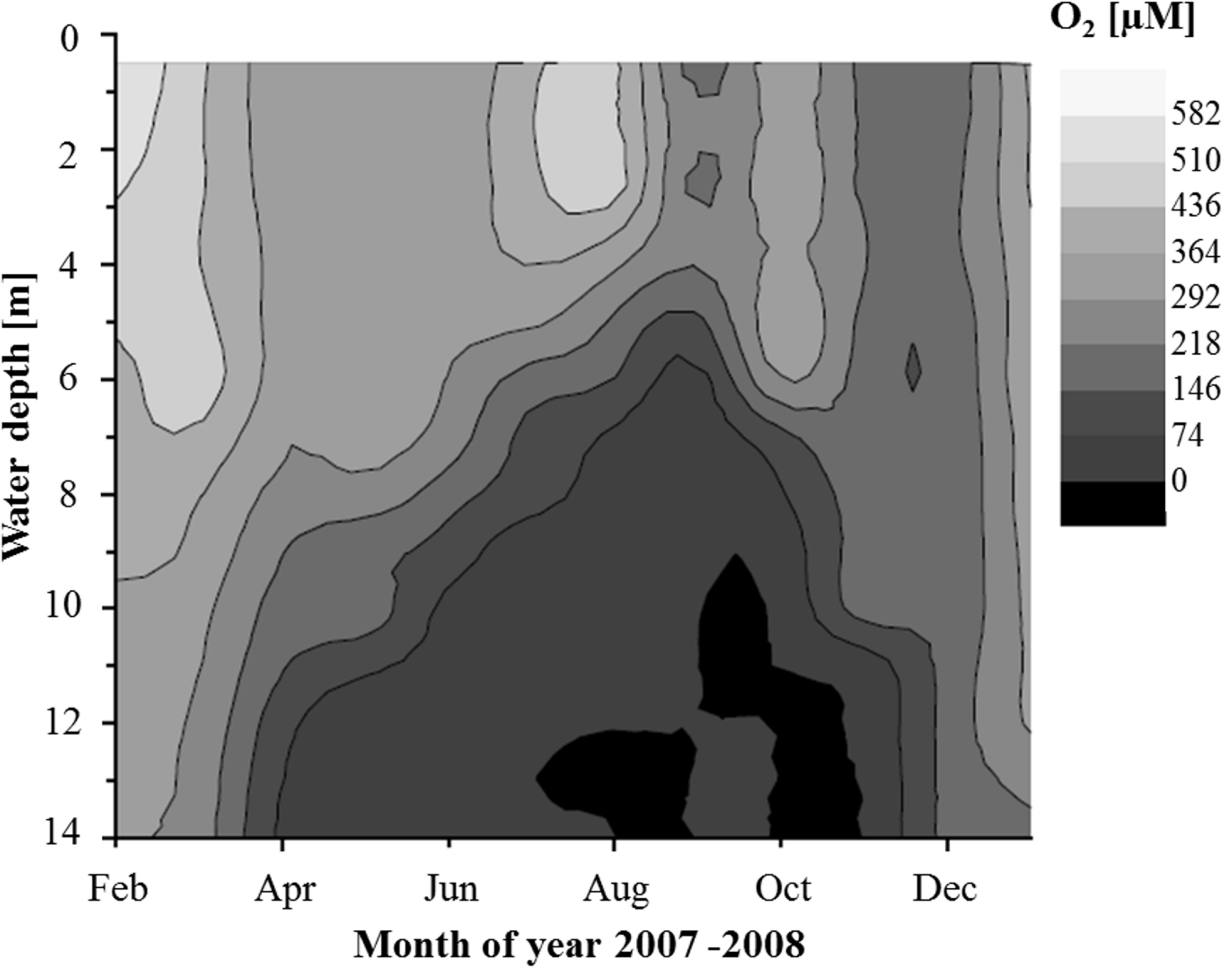
Oxygen concentrations in Lake Rotsee during an annual cycle (2007). Seasonal hypolimnic anoxia commenced in April and lasted until mid-November, when turnover occurred and the water column became fully mixed.

Lake Rotsee, situated near the city of Lucerne in Central Switzerland, is a small eutrophic sub-alpine lake. It is located at 436 m a.s.l., is 2.4 km long, 0.4 km wide and has an average depth of 9 m, with its deepest point being 16 m. The lake is monomictic and its waters circulate on a yearly basis. Depending on weather conditions, stratification commences as early as April and remains relatively stable until November (Fig 1). Bottom waters retain year round temperatures of 7°C and stratification promotes the formation of a chemocline located between 8–11 m depending on weather conditions [30]. The chemocline sets the boundary between the anoxic sulfidic hypolimnion and the oxic epilimnion.

### Sample collection

Sampling was carried out during August of 2012 and 2013. Samples were collected from a boat at the deepest part of the lake (47°04.259‘ N, 8°18.989‘ E). Conductivity, turbidity, depth (pressure), temperature and pH were measured with a multi-parameter probe (XRX 620, RBR). Dissolved oxygen was recorded online with two micro-optodes with detection limits of 125 nM (normal) and 20 nM (trace) [31] (types PSt1 and TOS7, PreSens). Samples for Winkler titration were taken at 2 m depth in order to verify the accuracy of the oxygen sensors. Photosynthetically active radiation (PAR) was measured with a spherical quantum sensor (LI-190 SB, LI-Cor) connected to a quantum meter (LI-188, LI-Cor). In parallel, a second quantum sensor recorded surface radiation. Water samples for nitrate (NO_3_^-^), nitrite (NO_2_^-^), ammonium (NH_4_^+^), sulfate (SO_4_^2-^), sulfide (HS^-^), dissolved inorganic carbon (DIC) and metal species (Fe and Mn) were taken with a rosette syringe sampler equipped with 12 syringes (60 ml) [31], which were triggered online at the appropriate depths. Samples taken with the syringe sampler were immediately distributed into vials containing the appropriate fixative if necessary. 15 ml were filtered (0.22 μm pore size) into tubes for NO_3_^-^, NO_2_^-^, NH_4_^+^ and SO_4_^2-^ analyses. Samples for HS^-^ determination were added to zinc acetate (final concentration ~1.3%). For dissolved (< 0.45 μm) and total metal fractions, water was acidified immediately with Suprapur HNO_3_ to a final concentration of 0.1 M. All other samples were collected with a Niskin bottle (5 l) from which water was dispensed through rubber tubing and bottles filled carefully avoiding bubbling or shaking and allowing water to overflow before sealing. To determine CH_4_ concentrations, 120 ml serum bottles were filled without headspace and bubbles, preserved with NaOH (pH > 12), closed with butyl rubber stoppers and sealed with metal crimps. Samples for catalyzed reporter deposition-fluorescence in situ hybridization (CARD-FISH) analysis were fixed immediately in the field with formaldehyde (2% [v/v] final concentration). Water for ^13^C-CH_4_ incubation experiments was collected in 160 ml sealed serum bottles or 1 l Schott bottles (closed with butyl rubber stoppers) without a headspace and kept cold and dark until further processing.

### Analytical techniques

Nitrate, sulfate and ammonium were measured on the same day as sampled by ion chromatography (881 Compact IC pro and 882 Compact IC plus, Metrohm). Nitrite (on the same day as sampled) and sulfide concentrations were determined by spectrophotometry as described by Griess [32] and Cline [33], respectively. A total organic carbon analyzer (TOC-L, NDIR detector, Schimadzu) was used to measure DIC concentrations. Dissolved and total fractions of Fe and Mn were analyzed by inductively coupled plasma-mass spectrometry (ICP-MS) (Element2, Thermo-Fisher).

For the determination of chlorophyll *a* concentrations, 700 ml of sampled water were filtered onto glass fiber filters (GF/F, Whatman). Subsequently chlorophyll *a* was extracted from the filters with 8 ml ethanol (90% [v/v], for 10 min at 75°C) [34]. Extracts were measured by high-performance liquid chromatography (HPLC) according to Murray et al. [35]. After isochromatic separation on a column (Merk LiChroCart 250–4; 1.0 ml min^-1^ flow rate, 49.5% methanol and 45% ethyl acetate), detection was done at 430 nm with a photodiode array detector (MD-2018 PLUS, Jasco).

Methane concentrations were determined by headspace injection [36] after creating a 25 ml N_2_ headspace. Following equilibration through sonication, CH_4_ concentrations were measured on a gas chromatograph (GC) (Agilent 6890N, Agilent Technologies) equipped with a Carboxen 1010 column (30 m x 0.53 mm, Supelco) and with a flame ionization detector (FID). Taking solubility constants for CH_4_ [37] into consideration, dissolved concentrations were calculated from the headspace data. Additionally, stable carbon isotopic ratios of methane were measured in the same headspace by isotope ratio mass spectrometry (IRMS). In a trace gas instrument (T/GAS PRE-CON, Micromass UK Ltd) injected gas samples were first purified (removal of CO_2_ and CO) by a series of chemical traps (magnesium perchlorate, Carbo-Sorb and Sofnocat) and a cold trap (liquid nitrogen), remaining CH_4_ was then oxidized to CO_2_ (in a combustion furnace) and concentrated (removal of N_2_O) by cryogenic freezing. Subsequently, the purified and concentrated sample was transferred to a connected mass spectrometer (GV Instruments, Isoprime), where isotopic ratios of the combusted CH_4_ were analyzed. Resulting ratios are given in conventional δ notation in comparison to the Vienna Pee Dee Belemnite (VPDB). Moreover, a 1% CH_4_ stock was measured intermittently between samples in order to check instrumental precision (~0.7 ‰) and correct for possible drifts.

### Chemical flux calculation

Chemical fluxes of the respective species were determined from the concentration profiles where gradients were highest. For O_2_, NO_3_^-^ and dissolved Mn they were calculated across the chemocline between 8–9 m depth. The flux of dissolved Fe was determined between 10–14 m. Assuming upwards diffusion from the sediment, fluxes of NH_4_^+^ and CH_4_ were also assessed between 10–14 m depth. Diffusive fluxes (*J*) were calculated using Fick’s first law:

$$J=-K_{z}\frac{\partial C}{\partial x}.$$ (1)

where *K*_*z*_ denotes the vertical turbulent diffusion coefficient, *C* is the concentration and *x* is the depth range. The concentration gradient was determined by linear regression. Diffusion coefficients in lakes have been estimated to range between 0.012–1.16 cm^2^ s^-1^ [38]. According to values (0.01–0.04 cm^2^ s^-1^), which have been typically used for flux calculations and modeling at low turbulence levels like in Lake Rotsee [39,40,41], we used a diffusion coefficient on the lower end of 0.01 cm^2^ s^-1^. This value was also determined from temperature profiles of Rotsee and utilized for flux calculations in Schubert et al. [30]. For this study conditions might have changed, therefore absolute values are estimations, but relative proportions between solute fluxes are valid.

To determine the flux of electrons, the diffusive flux was multiplied by the number of electrons (e^-^) a particular species can donate or accept assuming complete oxidation or reduction (i.e. 8 e^-^ for the oxidation of CH_4_ to CO_2_). Electron equivalents served as an alternative way (other than concentration) to quantitatively express the difference between the amount of reductants and their potential oxidants.

### ^13^C-CH_4_ incubation experiments and methane oxidation rates

#### Methane oxidation rate incubations

Rate incubations were carried out with water retrieved from 8, 9, 10 and 11 m. Collected water was bubbled with a continuous flow of He for ca. 15 minutes to remove potential trace oxygen contamination introduced during sampling. Subsequently, all bottles were amended with 5 ml of a saturated 50 at.% ^13^C-CH_4_ (99%, Campro Scientific) solution (prepared with sterile, anoxic Nanopure water), which resulted in a final concentration of ~50 μM CH_4_ in each incubation. After this, two bottles received no further additions and served as the light and dark conditions setups. A third bottle received 15 μM O_2_ (final concentration) from an O_2_ saturated stock solution (prepared with sterile Nanopure water). A fourth bottle received a treatment of 3-(3,4-dichlorophenyl)-1,1-dimethylurea (DCMU); dissolved in 96% ethanol (EtOH) to a final concentration of 10 μM. A fifth bottle was supplemented with 96% EtOH with the same volume (0.15 ml) as the DCMU addition. DCMU was applied as a photosynthesis inhibitor [42] and EtOH as a control setup to test for its possible toxic effects on the microbial community. Water from individually amended bottles was then distributed into 12 ml Exetainers (Labco Ltd) according to the procedure described by Holtappels et al. [43] and incubated at 6°C in either dark (dark and O_2_ setups) or light conditions (~10 μE; light, DCMU and EtOH setups). At each sampled time point an Exetainer was preserved with 200 μl zinc chloride [50% (w/v)] and stored at room temperature (RT) until analysis. CH_4_ oxidation was measured as the production of ^13^C-CO_2_. For this, 2 ml of sample were transferred into 6 ml Exetainers, headspace was exchanged with He and samples were acidified with the addition of ~100 μl concentrated H_3_PO_4_. ^13^C-CO_2_, which outgassed from the liquid phase, was subsequently measured by gas chromatography—isotope ratio mass spectrometry (GC-IRMS) (Fisons VG Optima).

In order to determine concentrations of produced ^13^C-CO_2_, δ^13^C-CO_2_ (‰) was first converted to a fractional abundance (FCO2C13):

FCO2C13=AR×(δC13CO21000+1)1+AR×(δC13CO21000+1). (2)

where *AR* is the absolute ratio of mole fractions of carbon (0.0111796) [44]. Relative differences in fractional abundance were then calculated between the beginning of the experiment (0 d) and each subsequent time point. The differences were converted to concentration of CO_2_ by multiplying with the in situ DIC concentration of the respective sampling depth. Taking into account that the addition of ^13^C-CH_4_ only renders a potential or maximum rate, methane oxidation rates were estimated from the slopes of linear regression of ^13^C-CO_2_ production over the time interval of incubation of 7 d in 2013 (2 d in 2012). When the production of ^13^C-CO_2_ was non-linear, an initial rate was calculated in some instances. This was determined by taking the change in produced ^13^C-CO_2_ over the steepest segment of the time series and dividing this by the corresponding number of days.

#### Growth and activity incubations

Bulk incubations were performed with water retrieved from 9 and 11 m. The purpose of these experiments was to monitor cell growth and cellular ^13^C uptake. For each depth eight 160 ml sterile serum bottles were each filled with 120 ml water in an anoxic (N_2_) glove box (Iner Tec). Bottles were sealed, crimped and amended with methane (50 at.% ^13^C, ca. 1.8 mM CH_4_ in the water phase). Each experimental setup was prepared in duplicate bottles; thus four bottles from each depth received no further addition and served as the dark and light setups. Duplicate bottles were supplemented with O_2_ (from an O_2_ saturated stock solution prepared with sterile Nanopure water) to a final concentration of ~15 μM and another two bottles were amended with 10 μM DCMU. All bottles were then incubated at 6°C in either dark (dark and O_2_ setups) or light (~10 μE; light and DCMU setups) conditions for a duration of 11 d. Periodically, 2 ml samples for ^13^C-CO_2_ measurements were withdrawn anoxically, transferred into 6 ml Exetainers, fixed with zinc chloride and kept at RT until analysis by GC-IRMS as described above. After 2, 7 and 11 d of incubation, 5 ml of water was removed and fixed with formaldehyde (see above for details) for CARD-FISH and nanometer-scale secondary ion mass spectrometry (nanoSIMS) analyses. In addition, at the end of the incubation the remaining water was filtered onto glass fiber filters (GF/F, Whatman) for bulk ^13^C uptake measurements performed with an elemental analyzer (Flash EA 1112, Thermo Scientific) coupled to an IRMS (Finnigan Delta Plus XP, Thermo Fischer Scientific).

### Catalyzed reporter deposition-fluorescence in situ hybridization

Water samples were fixed with formaldehyde overnight at 4°C before being filtered onto 0.2 μm polycarbonate GTTP filters (Millipore). Aliquots also intended for nanoSIMS were filtered onto Au/Pd sputtered filters of the same type. Filters were dried and stored at -20°C until processing. CARD-FISH was carried out according to Pernthaler et al. [45] with oligonucleotide probes. Cells were permeabilized with lysozyme (10 mg ml^-1^, for 1 h at 37°C) or proteinase K (15 μg ml^-1^) and sodium dodecyl sulfate (SDS) (0.5% [v/v]) for 3 and 10 min at RT, respectively. Bleaching of endogenous peroxidase activity was completed with 0.1 M HCl for 10 min at RT. Hybridization was performed for 2.5 h at 46°C with the respective formamide concentrations in the hybridization buffer and oligonucleotide probes labeled with horseradish peroxidase (HRP) (Biomers, Germany) (for details see S1 Table). Probes Mgamma84 and -705 and AAA-FW-641 and -834 were used in a 1:1 mix. After hybridization, probe signal was amplified with the tyramide Oregon Green 488 (1 μl ml^-1^) for 30 min at 37°C. Filter pieces were counterstained with 4',6-diamidino-2-phenylindole (DAPI, 1 μg ml^-1^) and embedded in a 4:1 mix of Citifluor/Vectashield and mounted onto glass slides. Cell numbers were quantified with a grid ocular of an epifluorescence microscope (Axioskop 2, Zeiss) by counting 20 randomly selected fields of view. Proportions of the different groups were calculated based on total cell numbers, which were enumerated with DAPI. Probe NON338 was used as a negative control.

### Nanometer-scale secondary ion mass spectrometry

Fixed water samples filtered onto Au/Pd-sputtered polycarbonate filters were hybridized with Mgamma84 and -705 probe mix as described above. Afterwards, circular pieces (5 mm diameter) were cut out and separate fields of view containing 2–3 hybridized cells were marked in a laser micro-dissection microscope (LMD) (DM 6000 B, Leica Microsystems). Filter circles were then embedded in Citifluor/Vectashield and mounted onto glass slides to be viewed in an epifluorescence microscope (AxioPlan, Zeiss). Finally, filter pieces were rinsed with 96% ethanol, dried and loaded into the nanoSIMS sample holder.

Laser-marked filter pieces were then analyzed with the nanoSIMS 50L (Cameca) at the Max Planck Institute for Marine Microbiology in Bremen, Germany. After pre-implantation with a Cs^+^ primary ion beam of 300 pA, a primary Cs^+^ ion beam with a diameter of < 100 nm and a beam current between 1.0 and 1.5 pA was rastered over the sample area. Secondary ion images of ^12^C^-^, ^13^C^-^, ^12^C^14^N^-^, ^31^P^-^ and ^32^S^-^ were recorded simultaneously using 5 detectors together with the secondary electron (SE) image with an image size of 256 x 256 pixels and 512 x 512 pixels and a dwell time of 1 ms per pixel. The scanned regions had a size of 20 x 20 or 30 x 30 μm and for each up to 40 planes were recorded. For the light setup 12 cells in 6 fields of view and 10 cells in 4 fields of view were analyzed for the sample after 2 and 7 d of incubation, respectively. 11 cells in 6 fields of view were measured for the O_2_ (dark conditions) setup after 2 d of incubation. The recorded images from each area were corrected for a possible drift of the stage or source, accumulated and evaluated with Look@NanoSIMS [46]. After defining regions of interest (ROI) (i.e. gamma-MOB, identified based on the CARD-FISH hybridization signal) and processing, ratios of ^13^C/^12^C, ^32^S/^12^C, ^31^P/^12^C and ^31^P/^32^S were obtained simultaneously in numerical and image format. For this study, ratios of ^13^C/^12^C were utilized to calculate cellular C-CH_4_ assimilation and ^32^S/^12^C served to confirm that cellular material was measured.

### Biovolume and methane assimilation calculation

The biovolume of gamma-MOB was approximated by enumerating cells and assuming an average spherical cell diameter of 2 μm. Subsequently, volume was converted to biomass by applying a calibration factor of 6.4 fmol C μm^-3^ [47]. Rates of cellular methane assimilation (*a*_*cell*_) were then assessed by the following formula:

$$a_{cell}=\frac{{}^{13}C{⁄}^{12}C_{cell}\times B}{L_{{}^{13}C}\times t}.$$ (3)

where ^*13*^ *C/* ^*12*^ *C*_*cell*_ is the ^13^C/^12^C ratio of the single cells measured with nanoSIMS, *B* is the calculated biomass, $L_{{}^{13}\text{C}}$ is the ^13^C-CH_4_ labeling percentage and *t* the incubation time [48]. The doubling time for gamma-MOB was assessed based on ^13^C/^12^C ratios of the single cells (converted to at.%), the amount of ^13^C-label added to the incubation (50 at.%) and the incubation time (2 d).

## Results

### Physicochemical conditions in Lake Rotsee

**Fig 2:**
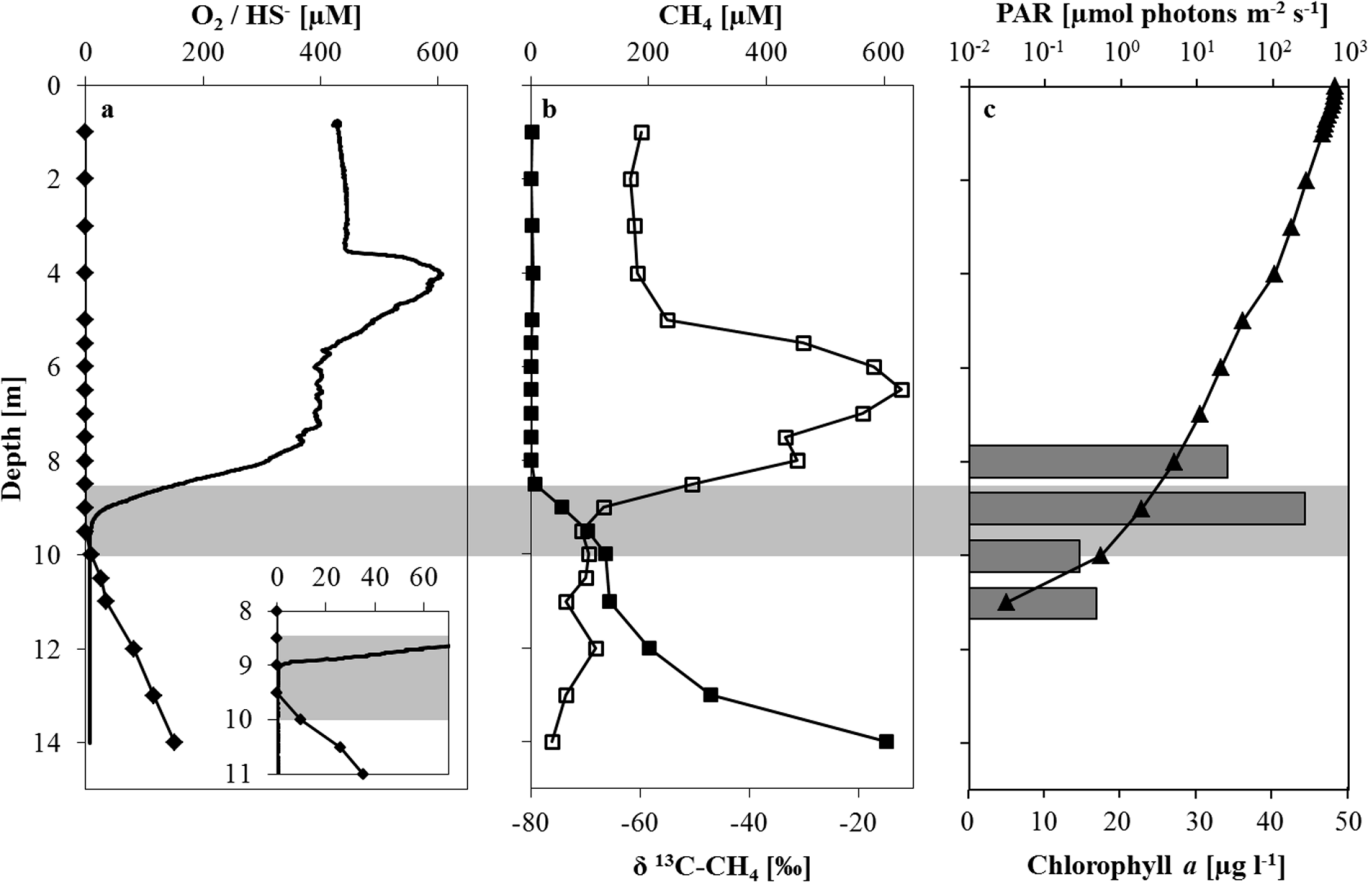
Physicochemical parameters in the Rotsee water column in August 2013. Depth concentration profiles of (a) oxygen (solid line) and sulfide (solid diamonds) with a detailed view from 8–11 m depth where the oxygen profile was recorded with a trace optode; (b) methane (solid squares) and methane carbon isotopes (empty squares); (c) photosynthetically active radiation (solid triangles) and chlorophyll *a* (grey bars). Note the logarithmic scale and the fact that PAR was recorded 2 d after all other sampling occurred. Grey shading denotes the location of the zone of methane oxidation.

In the dynamic water column of Lake Rotsee where anoxia prevails only during summer months the location of the oxic/anoxic boundary is variable (Fig 1). During the sampling campaign in August 2013 the oxycline was located at 9 m depth. The oxygen profile showed a decrease from 400 μM at the surface to below detection at 9 m and exhibiting a 600 μM maximum located at 4 m (Fig 2a). The downward flux of O_2_ was determined to be 101.3 ± 0.6 mmol e^-^ m^-2^ d^-1^. Sulfide concentrations increased below 9.5 m to a maximum concentration of 152 μM at 14 m depth. Methane concentrations decreased steadily from 600 μM near the sediment towards the oxycline. In the oxic epilimnion CH_4_ concentrations varied between 30 nM and 3 μM, never reaching the detection limit (~10 nM) (Fig 2b). Correspondingly, the δ^13^C of residual CH_4_ became progressively heavier from -76.2 ‰ at 14 m to -12.3 ‰ at 6.5 m, above which δ^13^C became lighter again. Based on the methane concentration and isotope profile we identified a zone of methane oxidation between 8.5 and 10 m. The calculated upward flux of CH_4_ was -104.3 ± 26.5 mmol e^-^ m^-2^ d^-1^. Light radiation based on total surface irradiation (22.9 E m^2^) reached 0.28% at the oxycline and was detected down to 11 m depth (0.01%) (Fig 2c). Nitrate concentrations did not change throughout the epilimnion (ca. 25 μM) and began declining at 8 m before disappearing at ~9 m (S1a Fig). Ammonium concentrations showed the opposite trend with concentrations increasing below 8 m to a maximum of ~360 μM at the sediment/water interface. Nitrite was not detectable throughout the water column (detection limit 1 μM). Sulfate concentrations decreased gradually from ~120 μM at the surface to ca. 50 μM at 14 m depth. Values for both dissolved Fe and Mn showed maximum concentrations of approximately 0.4 and 3 μM above the sediment, respectively (S1b Fig). Both metal species decreased towards the oxycline, where the decline of dissolved Fe was rather gradual and the Mn profile showed a sharp gradient at 9 m. Of the analyzed potential electron acceptors, oxygen and nitrate showed a decrease and dissolved (reduced) manganese an increase within the identified zone of methane oxidation. The total downward (O_2_ and NO_3_^-^) and upward (NH_4_^+^, Fe, Mn and CH_4_) fluxes were determined to be 112.5 ± 2.4 and -122.5 ± 26.7 mmol e^-^ m^-2^ d^-1^, respectively. In the case of ammonium we assumed oxidation to nitrate followed by immediate nitrate reduction to N_2_ due to no detectable nitrate production (i.e. 8–5 = 3 e^-^). Furthermore, we excluded SO_4_^2-^ and HS^-^ in the balance calculation as the former is probably first reduced in the sediment and the latter oxidized to S^0^ by phototrophic sulfur bacteria below the oxycline [49]. In 2012, O_2_ and CH_4_ concentration profiles showed similar trends but shifted upwards by 2 m, as the oxycline was located at 7 m instead of 9 m (S2a Fig). Concentrations of O_2_ and CH_4_ were in the same order of magnitude as in 2013, with maximum concentrations being 340 μM (at the surface) and 750 μM (at 14 m), respectively.

### Methane oxidation rates

**Fig 3:**
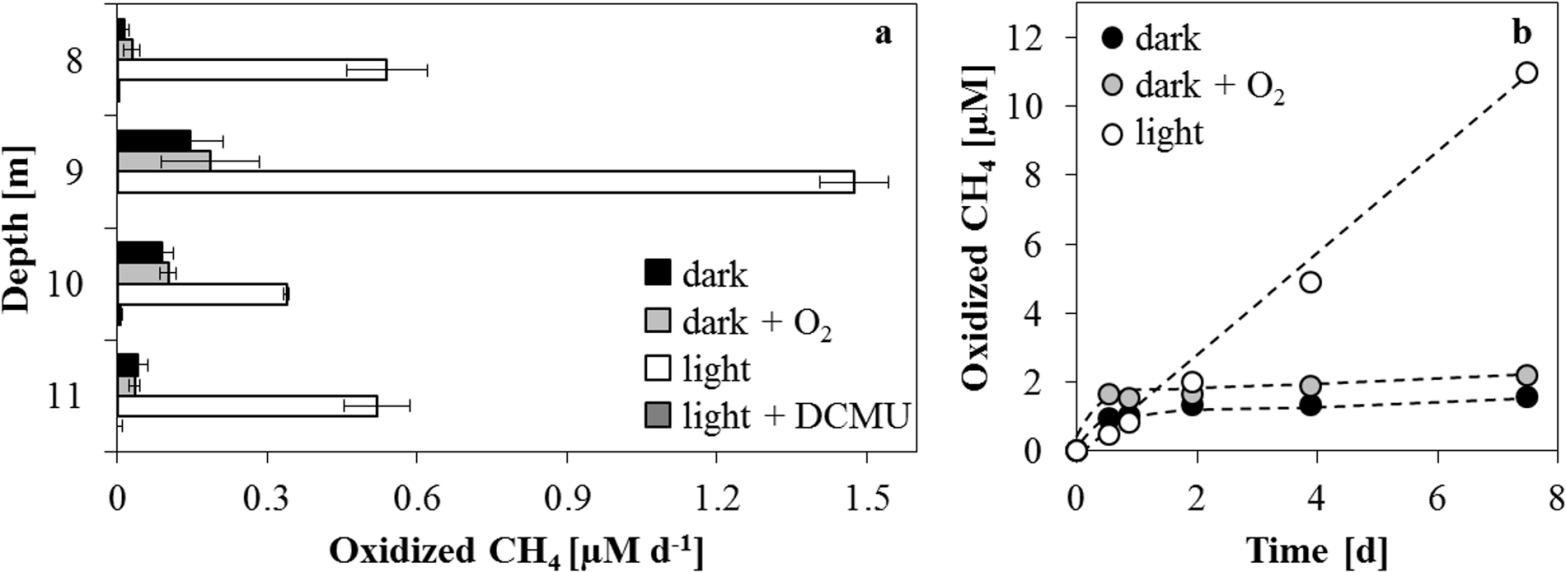
Methane oxidation rates at and below the oxycline. (a) Methane oxidation rates in different incubation setups from an oxic depth (8 m), the oxycline (9 m) and anoxic depths (10 m, 11 m). (b) Methane oxidation time series under dark (with and without added O_2_) and light conditions with water from 9 m depth. Note that the addition of oxygen resulted in higher initial methane oxidation rates which ceased during the course of the incubation whereas light treatment resulted in steady linear rates.

Methane oxidation potential was measured in laboratory incubations at depths, where the CH_4_ profile showed the steepest gradient and therefore the highest flux. The incubation depths represented oxic waters (8 m), the oxycline (9 m) and truly anoxic and sulfidic zones (10 and 11 m). Incubation under dark conditions, where only ^13^C-CH_4_ was added, yielded methane oxidation rates ranging between 15.4 nM d^-1^ (8 m) and 145.8 nM d^-1^ at the oxycline (9 m) (Fig 3a). At all incubated depths, additions of O_2_ (incubated in the dark) produced MO rates which were slightly higher than the corresponding dark setups, except at 11 m, where the O_2_ rate was lower. Interestingly, incubations from all depths resulted in higher MO rates when incubated in the light. Under light conditions measured rates were three- to 36-fold higher than in the corresponding dark incubations from the same depth. The highest stimulation in rates was observed at the shallowest depths (8 and 9 m) where the maximum rate reached 1.47 μM d^-1^. CH_4_ oxidation could not be detected in the killed controls or in the setups with additions of a photosynthetic inhibitor, DCMU. Along with the differing absolute rates, the kinetics of methane oxidation also varied among the different setups. Only incubation under light conditions yielded linear MO increase throughout the experiment (Fig 3b). Contrarily, under dark conditions, with or without added O_2_, an initial CH_4_ oxidation period of about one day was observed, which ceased subsequently. These distinct oxidation kinetics were evident at each incubated depth with plateau and linear trends always resulting from dark/O_2_ and light conditions, respectively. However, MO rates for all setups were comparable only if the first 24 hours were considered for calculation.

A similar light effect on MO oxidation rates was observed in incubations performed in 2012. Dark conditions in 2 d laboratory incubations from 8 m depth during the 2012 sampling campaign produced the lowest MO rate (0.38 μM d^-1^; S2b Fig). Whilst the addition of O_2_ caused an approximate doubling (0.74 μM d^-1^), light conditions produced a substantial increase in methane oxidation (1.51 μM d^-1^) compared to the dark setup. The MO kinetics for the different setups showed comparable plateau and linear trends as in 2013 (S2c Fig). Interestingly, light conditions only had an effect at 8 m depth, which represented anoxic conditions as the oxycline was located at 7 m. In contrast, at 6.5 m, immediately above the oxycline, light conditions had virtually no impact on the MO rates.

### In situ abundance of methanotrophic microorganisms

Total microbial cell numbers enumerated with DAPI approximated 3·10^6^ cells ml^-1^ at all incubation depths with the highest abundance reaching 3.8·10^6^ cells ml^-1^ at 11 m. The relative contribution of several microbial groups of interest was investigated using CARD-FISH with specific phylogenetic probes (S3 Fig). Bacterial cells (targeted by EUB338 I-III) showed a slight increase with depth, where the average amounted to ~65% of total DAPI counted cells. Archaeal abundance (as determined using the ARCH915 probe) was 4.2% of total cell counts at 8 m and decreased steadily to 2.4% at 11 m. The discrepancy between total cell numbers enumerated with DAPI and the sum of bacterial and archaeal abundance is possibly due to probes not covering all groups of present microorganisms. Targeted groups of anaerobic methane-oxidizing archaea (probes ANME-1-350 and ANME-2-538) or AOM-associated archaea (probe mix AAA-FW-641+AAA-FW-834) were not detectable at any of the investigated depths. In contrast, aerobic MOB, more specifically gamma-MOB (probe mix Mgamma84+Mgamma705), were found at all investigated depths below 8 m (Table 1), reaching highest abundances (1.6·10^4^ cells ml^-1^) in anoxic (sulfidic) waters at 11 m. Interestingly, gamma-MOB were not detected in the fully oxic water column, in agreement with very low methane concentrations at these depths. Alpha-MOB (probe Ma450) were not identified at any sampling depth during the 2013 sampling campaign. Contrarily, during the sampling campaign in 2012, the presence of both alpha- and gamma-MOB was confirmed at 6.5 and 8 m. Total cell numbers were of the same order of magnitude as in 2013 and on average alpha- and gamma-MOB constituted 1.9 and 0.6% of total DAPI counts, respectively.

**Table 1:** Abundance of aerobic gamma-methanotrophs. Total in situ cell abundance of gamma-MOB (targeted with the Mgamma 84+705 probe mix) along with their percentage of DAPI counted cells for all incubation depths (August 2013). Given errors denote the standard error of the mean of counted fields of view (20).

Depth [m]	Gamma-MOB cell abundance [cells ml^-1^]	Proportion of DAPI counted cells [%]
8	7.91·10^3^ ± 1.76·10^3^	0.26 ± 0.06
9	1.58·10^4^ ± 2.02·10^3^	0.50 ± 0.07
10	1.02·10^4^ ± 1.21·10^3^	0.38 ± 0.04
11	1.63·10^4^ ± 2.18·10^3^	0.43 ± 0.06

### Growth and activity of gamma-MOB

**Fig 4:**
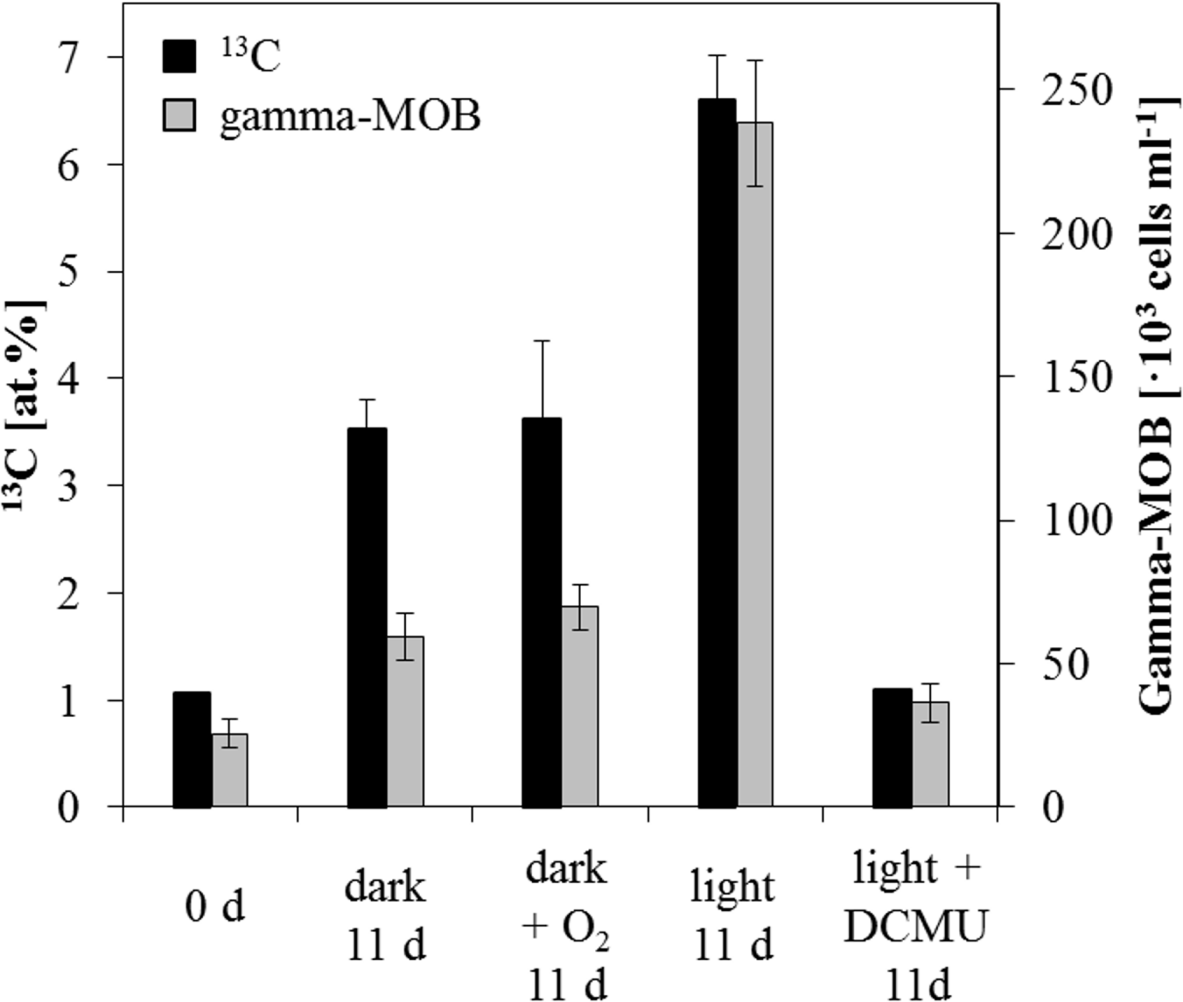
Gamma-MOB abundance and bulk ^13^C-uptake at the oxycline. Increase in ^13^C at.% enrichment (black) after 11 d of incubation for all performed setups. Error bars represent differences between triplicate measurements from duplicate incubations. Abundance of the gamma-MOB (grey) at the beginning (0 d) of the 9 m bulk incubation along with their cell numbers after 11 d of incubation in dark and light conditions. Error bars signify the standard error of the mean of counted fields of view (20). Note the two-fold higher uptake of ^13^C-CH_4_ in the bulk microbial biomass corresponding to the ~two-fold increase in gamma-MOB abundance under light conditions.

Since all aerobic MOB assimilate at least part of the methane they oxidize [50], we could trace growth and activity of the gamma-MOB by using ^13^C-CH_4_ as a tracer during the incubations. Total cell counts of the gamma-MOB and the uptake of ^13^C-CH_4_ by bulk and single-cell analyses were monitored at the beginning and end of the incubations. Corresponding to the highest MO rates, incubation under light conditions from 9 m depth (oxycline) resulted in the highest, nearly ten-fold increase of the gamma-MOB, from 2.6·10^4^ to 2.4·10^5^ cells ml^-1^ (Fig 4). In comparison, the dark incubation from the same depth only resulted in a ca. two-fold increase compared to initial cell numbers. This apparent fast growth of the gamma-MOB during the course of the light incubation coincided with a substantial enrichment of the bulk ^13^C content of the microbial biomass, which increased from 1.1 at.% at the beginning of the experiment (i.e. natural abundance) to 6.6 at.% after 11 d (Fig 4). Contrarily, under dark conditions measured ^13^C enrichment of the total biomass only reached 3.5 at.% and 3.6 at.% with the addition of O_2_, while no ^13^C uptake was detected in the DCMU treatment. The comparable bulk ^13^C enrichment in the dark setups mirrored the almost identical oxidation rates measured for these incubations. At 11 m, where stimulation of MO by light was less pronounced, there was no detectable difference in bulk ^13^C-uptake between the setups. Corresponding to this, gamma-MOB showed only a minor increase from 4.1·10^4^ cells ml^-1^ to 4.4·10^4^ and 7.8·10^4^ cells ml^-1^ under light conditions and with added O_2_, respectively.

**Fig 5:**
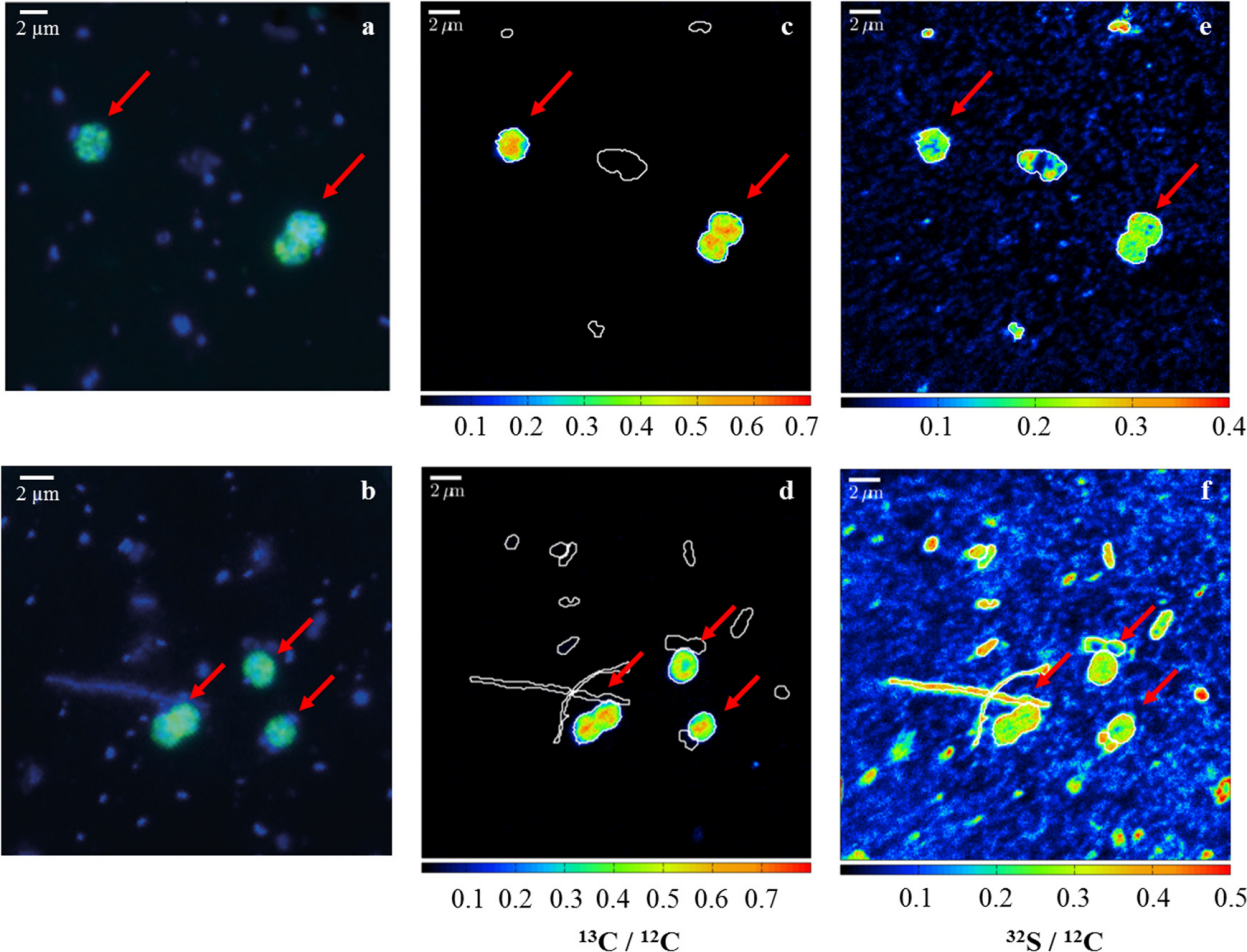
Methane assimilation by single gamma-MOB cells at the oxycline. (a, b) Gammaproteobacterial methanotrophs at the oxycline visualized by DAPI (blue) and fluorescence in situ hybridization with Mgamma 84+705 probes (green). Corresponding nanoSIMS images of (c, d) ^13^C/^12^C and (e, f) ^32^S/^12^C ratios after 2 d of incubation in light conditions (upper panel) and with supplemented oxygen (lower panel) from 9 m depth. Red arrows indicate selected hybridized cells. Scale bars represent 2 μm.

Single-cell analyses of samples incubated with ^13^C-CH_4_ in the light from 9 m depth showed that the abundant gamma-MOB were indeed strongly enriched in ^13^C (Fig 5 and S4 Fig) with cellular ^13^C/^12^C ratios averaging 0.40 ± 0.04 (27.6 ± 2.2 at.%) (S5 Fig) after 2 d of incubation. This corresponds to a calculated doubling time of approximately 1.8 days. Gamma-MOB cells incubated for the same time (2 d) with added O_2_ showed comparable cellular enrichment of 0.36 ± 0.05 (27.1 ± 2.0 at.%), accounting for approximately the same calculated doubling time. This corresponds to an average calculated cellular C-CH_4_ assimilation rate of 108 and 96 amol C d^-1^ for gamma-MOB under light conditions and with the addition of O_2_, respectively. Moreover, this matches measured MO rates from both setups, which were comparable during the first 2 d of incubation. Measured cells (~120), which were not identified by CARD-FISH, had average ^13^C/^12^C ratios of 0.032 ± 0.025 (3.11 ± 2.42 at.%), probably resulting from secondary uptake of ^13^C-CO_2_ by autotrophy.

## Discussion

### Methane oxidation at the oxycline and below

During stable summer stratification highest MO potential was detected directly at the oxycline (9 m) and immediately below (10 m) in the Rotsee water column. This is based on measured in vitro MO rates as well as relevant in situ chemical parameters. The CH_4_ depth profile (Fig 2b) shows CH_4_ consumption between 8.5 and 10 m, where concentrations change most dramatically. The δ^13^C signature of residual methane corroborates microbial MO, as isotopic values became substantially heavier at 9 m, resulting from the preferential uptake of the lighter isotope by microorganisms leaving a CH_4_ pool enriched in ^13^C [51]. Interestingly, the isotopic signature again shifted towards lighter values in the epilimnion suggesting a local source supplying CH_4_ to the water column at this depth, besides methanogenesis occurring in the sediments of the hypolimnion. It is feasible that isotopically light CH_4_ was laterally transported from littoral sediments [52]. Alternatively, methanogenesis occurring in the oxic epilimnion could also be the source of the this isotopic shift [53].

Comparison of Rotsee MO potential resulting from the same treatments at different depths, shows that maximum CH_4_ oxidation occurs at the oxycline. As reported by Schubert et al. [30], oxidation rates for Rotsee peaked within the oxycline at ~5 μM d^-1^, which is higher than what was measured in this study. However, this discrepancy in determined rates could be attributed to distinct methods being used, namely ^3^H-CH_4_ and in situ CH_4_ [30] versus ^13^C-CH_4_ in this investigation and also from temporal changes in conditions and the microbial community at those times. Potential oxidation rates (addition of ^14^C-CH_4_ or ^3^H-CH_4_) measured for other lake systems are variable, but generally most efficient CH_4_ consumption is located within the oxic/anoxic interface where gradients of O_2_ and CH_4_ are highest [21,54]. In smaller seasonally stratified lakes maximum rates occur within the oxycline or suboxic regions and reach similar magnitudes [54,55]. Methane consumption reported for deeper lake systems with varying conditions are also on the same scale with maximum MO potential corresponding to completely anoxic regions [27]. Additionally, fully oxygenated conditions can even inhibit CH_4_ oxidation [56], which likely explains observations of less efficient MO in oxic epilimnia [27,54,57]. This agrees well with our findings in Lake Rotsee, where methane oxidation, is also proceeding below the oxycline in anoxic (10 and 11 m) zones at rates that are even higher than under oxic conditions (8 m).

Oxidation below the oxycline, under apparently anoxic conditions, hints at AOM taking place. The onset of fully anoxic conditions and thus the possibility of AOM were confirmed by the precise detection of the oxic/anoxic transition. Micro-optodes with a detection limit of 20 nM and a response time of 7 s [31] allowed for the exact identification of the oxycline at 9.05 m on the particular sampling day (August 2013). The location of the oxycline in Lake Rotsee can however be quite variable, sometimes changing on a daily or even hourly basis. Multiple O_2_ profiles taken throughout the sampling day, confirmed a depth variability reaching down to 9.4 m. However, water taken from 10 and 11 m represented oxygen free conditions at the time of sampling. Consequently, these findings suggest that methane is oxidized anaerobically below the transition zone with an electron acceptor other than O_2_ or that a temporal supply of oxygen below the oxycline exists, which is not detectable by the methods utilized herein.

### Stimulation of methane oxidation by light

Other electron acceptors (NO_3_^-^, NO_2_^-^, Fe(III), Mn(IV) and SO_4_^2-^) supporting AOM could play a role at and below the oxycline. However, when taking concentrations of alternative oxidants and the molar ratios required for their involvement in CH_4_ oxidation into account, it is unlikely that they contribute significantly below the oxycline. The only electron acceptor which was present in higher concentrations is SO_4_^2^. However, the flux of SO_4_^2-^ (~7.0 ± 1.5 mmol e^-^ m^-2^ d^-1^) across the CH_4_ consumption zone (8.5–10 m) is too low to sustain CH_4_ oxidation. Additionally, SO_4_^2-^ and HS^-^ profiles did not show any regions of depletion or production in the water column, indicating that their distribution is diffusion controlled [49]. The possibility of cryptic sulfur cycling supplying SO_4_^2-^ for AOM via HS^-^ oxidation by reduced Fe or Mn [23,58] also seems unlikely considering this. HS^-^ is most likely completely oxidized by phototrophic sulfur bacteria below the oxycline [49] and therefore not available for other oxidation reactions. In line with this, no ANME or ANME-associated archaea, which mediate SO_4_^2-^-dependent AOM, were found. Therefore, it is unlikely that AOM is occurring at the depths investigated here and O_2_ appears to be the only relevant oxidant.

A source of O_2_ below the oxycline could be a result of oxygenic phototrophy under low light conditions, as this appears to stimulate CH_4_ oxidation at all incubated depths in Lake Rotsee. In 2013 dark incubations produced time series that showed an initial oxidation period (~1 d), after which a plateau with no further oxidation was reached (Fig 3b). A similar trend was observed with the addition of O_2_, where oxidation also ceased after a short period. Subsequently, no further oxidation was observed indicating that MO is not constant without a continuous supply of oxygen. Contrarily, light conditions produced rates that were at least three times higher than corresponding dark or supplemented O_2_ incubations. When also considering the corresponding time series, it becomes even more evident that light promotes continuous, constant CH_4_ consumption. Light conditions appear to have maintained MO potential and involved microorganisms apparently had access to all necessary substrates. In contrast, dark and O_2_ setups reached a plateau in oxidized methane quickly (after ~1 d) suggesting that the initially present electron acceptor was depleted after this short time. Since CH_4_ was never limiting in the incubation, the availability of O_2_ must have been restricting continuous oxidation. The discontinuation of MO could also be attributed to O_2_ being consumed for biological respiration or for the abiotic oxidation of other reduced species (i.e. Fe^2+^, HS^-^). Initial oxidation rates from most depths calculated during the first day of incubation were almost identical for the O_2_ treatments and non-amended samples, both incubated in dark conditions, except at 9 m. Here the O_2_ setup resulted in an initial rate (1.73 μM d^-1^), which was higher than the corresponding dark (1.14 μM d^-1^) or light incubation (1.47 μM d^-1^). This supports the notion that O_2_ is rate limiting at least at 9 m. However, even though 15 μM of O_2_ were supplemented, only 1.4 μM of CH_4_ were oxidized during the 7 d incubation period. Theoretically there should have been enough O_2_ to oxidize a total of ~7.5 μM of CH_4_. This fact along with amplified CH_4_ turnover and linear reaction kinetics under light conditions indicates that light exposure is the essential factor for MO at and also below the oxycline in Rotsee. Even though the light effect was also observed at 8 and 11 m, CH_4_ oxidation rates were substantially lower. As in situ methane concentrations were low (~1 μM) at 8 m and there was very low light penetration at 11 m (Fig 2c), it is likely that the microbial community was less well adapted to support efficient MO leading to lower determined rates. The mechanism of light stimulation was evident and observed during two successive years, implying that (1) this is a reoccurring rather normal process during stratification, (2) it has the potential to reestablish itself after lake turnover, (3) it is hence a relevant process for innumerable seasonally stratified lakes worldwide.

### Type I methanotrophs contribute actively to methane turnover

Both alpha- and gamma-MOB were detected in the Rotsee water column, however, alpha-MOB were only present in 2012. In fact, type I (gamma-) MOB are usually more abundant in lake water columns, which suggests that they play a more significant role in methane cycling [9,59,60]. This also appears to be the case in Lake Rotsee, where the consistent appearance of gamma-MOB, also in 2007 [30], indicates that they are an important member of the methane-oxidizing community. Even though gamma-MOB constituted only a small percentage of total cell numbers, the maximum of 0.5% corresponded to the location of the oxycline, where MOB are frequently found [61,62]. Studies based on molecular techniques indicate that type I (gamma-) MOB usually make up <5% of the total bacterial population [55,60]. This is in line with our findings and also supported by what was found previously for the Rotsee oxycline with gamma-MOB making up 2% of the biomass in 2007 [30]. Gamma-MOB comprised about 0.5% of total cell counts on our two consecutive sampling campaigns, indicating that gamma-MOB make up a stable proportion of the bacterial volume.

Interestingly, we found both types of MOB, which are obligate aerobic microorganisms, not only in oxygenated zones, but also in depths depleted of O_2_. This is not a novel observation as they have also been detected in completely anoxic waters in other lakes [27,28,60] and aerobic MOB appear to be able to withstand a lack of oxygen for an extended period of time [9]. It has also been speculated that MOB found in anoxic zones could simply have sedimented down from oxic waters and are no longer active [60]. However, their presence at depths devoid of O_2_ and their ability to survive anoxic conditions, could also indicate that intermittent sources of O_2_ could be available, i.e. from down-welling of cold oxygenated water [27]. In Lake Rotsee the presence of aerobic MOB corresponded to ongoing CH_4_ oxidation, thus inactivity of cells can be excluded and the presence of oxygen must be considered.

Along with their presence in anoxic waters, gamma-MOB also increased during the light incubation by nearly a factor of ten (Fig 4). In the dark, they increased ca. two-fold, similar to all other microbial groups investigated by CARD-FISH. The fact that highest MO rates corresponded to favorable growth of gamma-MOB suggests that they are responsible for the measured CH_4_ turnover. Direct evidence that the abundant gamma-MOB were actively involved in methane oxidation comes from nanoSIMS imaging. ^13^C/^12^C ratios recorded for gamma-MOB were about 40 times higher (27.6 at.% on average) than natural abundance values (Fig 5 and S4 Fig). Based on these recorded ratios ^13^C assimilation per cell was determined to be 108 amol C d^-1^, which corresponds to a doubling time of 1.8 d for gamma-MOB. Since this is also the time needed for the population to reach the ^13^C-labeling percentage, average enrichment per cell did not show further increase after this time. Gamma-MOB in the O_2_ setup were enriched to almost the same degree, indicating that they also assimilated C during this time period, which corresponds to a comparable doubling time (1.9 d) and initial MO rates resulting from the two treatments. Comparable ^13^C assimilation and doubling times were reported for gamma-MOB in Lago di Cadagno, where uptake rates were in the fmol C d^-1^ per cell range [28]. Doubling times reported for pure cultures of type-I MOB are usually on a time scale of a few hours [63,64], however this represents growth under optimal conditions and is therefore not easily compared to environmental settings. Additionally, it must be considered that the gamma-MOB did not derive all of their cellular C from methane, but also from the unlabeled DIC pool, which could result in an underestimation of the doubling time. Our results show that gamma-MOB contribute substantially to methane turnover in Lake Rotsee, although the possible involvement of other unidentified methane-oxidizers cannot be excluded.

### Photosynthesis fueled aerobic methane oxidation

Light stimulated methane oxidation at and below the oxycline of Lake Rotsee along with active aerobic gamma-MOB in anoxic regions hints at a light driven supply of O_2_. Methane oxidation did not occur in incubations spiked with DCMU, a photosynthetic inhibitor. This along with enhanced oxidation rates and linear reaction kinetics under light conditions (Fig 3) indicates that CH_4_ oxidation could only be sustained when photosynthesis was occurring. A strict dependence of oxidation on photosynthesis was also apparent when considering that the difference in initial oxidation rates between the dark and O_2_ setups was 0.6 μM d^-1^ at most, which suggests that the addition of O_2_ (15 μM) had virtually no effect. Moreover, light-dependent CH_4_ oxidation rates, chlorophyll *a* concentrations and gamma-MOB cell abundance nicely corresponded to each other (Figs 3 and 2c and Table 1). This suggests that potential CH_4_ oxidation rates were dependent on chlorophyll *a* concentrations and MOB abundance. In situ primary production rates (S2 Table) show that O_2_ can be generated at and below the oxycline. The rates of photosynthetic O_2_ production were in the same range as those of MO and depending on the depth could account for 90 to 100% of the measured methane oxidation rates, even though one should keep in mind that produced O_2_ is also available for consumption by other aerobic organisms. Additionally, laboratory incubations only yield a CH_4_ oxidation potential, as defined concentrations of CH_4_ were added, which might not have mirrored in situ conditions at some depths. However, the fact that both processes were occurring on the same scale and at the same depths, provides compelling evidence for a direct link. Light radiation, which was measured 2 d after all other profiles were taken, was detected down to 11 m depth (Fig 2c). Though we cannot be absolutely certain, it is probable that light reached even deeper on the main sampling day, as it was sunny, whereas it was cloudy when the PAR profile was recorded. Moreover, from measured potential MO rates, chlorophyll *a* concentrations and the presence of gamma-MOB, it is likely that this scenario could be taking place if light conditions were optimal at all incubated depths. Considering this, photosynthetic O_2_ production coupled to CH_4_ oxidation has the potential to proceed not only at the oxycline but also in apparently anoxic depths. Besides photosynthesis supplying oxygen, the possibility of an alternative source, i.e. from flood events introducing cold oxygenated water into otherwise anoxic zones, cannot be excluded completely [65]. However, since there were no extreme weather events before or during either of our sampling campaigns, this seems relatively unlikely. Furthermore, Lake Rotsee is surrounded by hills protecting it from strong winds, thus impeding disturbances in stratification due to wind forcing.

Thus we propose that aerobic CH_4_ oxidation was taking place throughout incubated depths and that photosynthetically generated O_2_ was consumed by gamma-MOB at and below the oxycline. This direct coupling between photosynthesis and methane oxidation could also explain supposed AOM proceeding via alternative oxidants in Lake Rotsee concluded by Schubert et al. [30]. At least during summer stratification aerobic CH_4_ oxidation fueled by oxygenic photosynthesis appears to be the dominant process and observed during two consecutive years. Since produced O_2_ would have been consumed immediately, this lead to oxidation rates being measured in seemingly anoxic regions and possibly mistaken for AOM previously.

### Implications for methane oxidation in shallow stratified water bodies

A similar scenario with aerobic CH_4_ oxidation depending on photosynthesis derived oxygen was also observed in Lago di Cadagno reported by Milucka et al. [28]. Lago di Cadagno, an alpine lake situated in Southern Switzerland, is a meromictic lake stabilized by sulfate-rich intrusions [66]. It is relatively small covering an area of 0.26 km^2^ and does not undergo seasonal mixing [66]. Systems such as Lago di Cadagno are scarce, as permanent stratification is usually an attribute of very deep lakes where the accumulation of dissolved species causes a density difference between the epi- and hypolimnion preventing complete mixing [67]. Our results indicate that the link between photosynthesis and aerobic methane oxidation is not only restricted to meromictic systems, but also occurs in seasonally stratified lakes. In fact the majority of lakes in temperate, subtropical and high elevation climatic zones are governed by mixing schemes, with dimictic (circulation twice per year) being the most prevalent [29]. Monomictic lakes, such as Lake Rotsee, are widespread in temperate regions [29]. Additionally, systems with a similar size as Rotsee (~1 km^2^) and smaller comprise about 54% of the global lake surface area [2] and most either circulate on a yearly or biyearly basis. Since most of these systems are also likely to be shallow with deep light penetration compared to their maximum depth, they might also host aerobic CH_4_ oxidation coupled to oxygenic photosynthesis. In cases were light reaches fully anoxic regions, elevated MO rates could therefore be mistaken for AOM.

This investigation also demonstrates the adaptability of the microbial community within such a variable system. The interrelation between oxygenic phototrophs and aerobic methanotrophs appears to be reproduced on a yearly basis emphasizing the ability to rebound after a mixing event. Shifts in the methanotrophic community according to O_2_ and CH_4_ concentrations have also been observed on a daily basis in a shallow floodplain lake [68]. Here we show that variation in the MOB structure might not only depend on substrate fluxes, but also on microbial interactions over much broader time scales, which have gone mostly unnoticed to date and could play an essential role in the majority of lacustrine environments.

The oxic/anoxic transition and the zone below probably represent the most suitable location for methane oxidation [27,54,57], as both high light intensity [69,70] and oxygen concentrations [56] in the epilimnion are likely to inhibit MO. Therefore, processes occurring in this region are probably also more relevant for methane removal in limnic systems. Assuming that Lake Rotsee is an adequate proxy for lakes of its size, this metabolic interaction between phototrophs and methanotrophs could be an additional important mechanism diminishing the CH_4_ flux into the atmosphere.

## Supporting Information

S1 Fig Profiles of dissolved ions in the Rotsee water column in August 2013. (a) Concentration depth profiles of nitrate (solid diamonds), ammonium (empty diamonds) and sulfate (solid triangles); and (b) dissolved iron (solid circles) and dissolved manganese (empty circles). The grey shading denotes the zone of methane oxidation.
(TIF)

S2 Fig Chemical profiles and methane oxidation rates in August 2012. (a) Concentration depth profiles of oxygen (solid line) and methane (solid squares). (b) Methane oxidation rates from 6.5 and 8 m depth in dark conditions, with the addition of O_2_ (in the dark), in light conditions and the killed control. (c) Incubation time series from 8 m depth in the dark, with supplemented O2 (in the dark) and in the light. Bulk methane turnover (b) and oxidation time series (c) from 8 m depth were measured in the same incubation.
(TIF)

S3 Fig In situ cell abundances of different microbial groups at various water depths in Lake Rotsee in August 2013. Total cell numbers of DAPI counted cells, bacteria (probe mix EUB338 I-III), archaea (probe ARCH915), alphaproteobacteria (probe alfa968) and gamma-MOB (probe mix Mgamma84+705) at incubation depths. Error bars denote the standard error of the mean of counted fields of view (20). Note the logarithmic scale.
(TIF)

S4 Fig Methane assimilation from analyzed setups from 9 m depth. Fluorescent micrographs of gamma-MOB (a-c) visualized by DAPI (blue) and in situ hybridization with Mgamma 84+705 probes (green). Corresponding nanoSIMS images (d-f) of ^13^C/^12^C and (g-i) ^32^S/^13^C ratios after 2 d in light conditions (upper panel), after 2 d amended with oxygen (middle panel) and after 7 d in light conditions (lower panel). All scale bars are 2 μm and red arrows indicate hybridized/measured cells.
(TIF)

S5 Fig Methane assimilation by gamma-MOB at the oxycline of Lake Rotsee. ^13^C-CH_4_ uptake (represented as ^13^C/^12^C ratios) by single gamma-MOB in the light incubation (after 2 d, n = 12 cells and 7 d, n = 10 cells) and with supplemented O2 (after 2 d, n = 11 cells).
(TIF)

S1 Table CARD-FISH oligonucleotide probes applied to Rotsee samples. HRP probes along with their specificity, applied permeabilization, % [v/v] formamide in the hybridization buffer and reference.
(PDF)

S2 Table Primary production rates measured in August 2013. Primary production rates were quantified at in situ conditions 2 d after all other physicochemical profiles were taken and methane oxidation rate experiments were set up.
(PDF)
