# Supplementary figures and images for: Light-Dependent Aerobic Methane Oxidation Reduces Methane Emissions from Seasonally Stratified Lakes
Source: PLoS One. 2015 Jul 20;10(7):e0132574. doi: 10.1371/journal.pone.0132574 (PMC4508055; doi:10.1371/journal.pone.0132574)

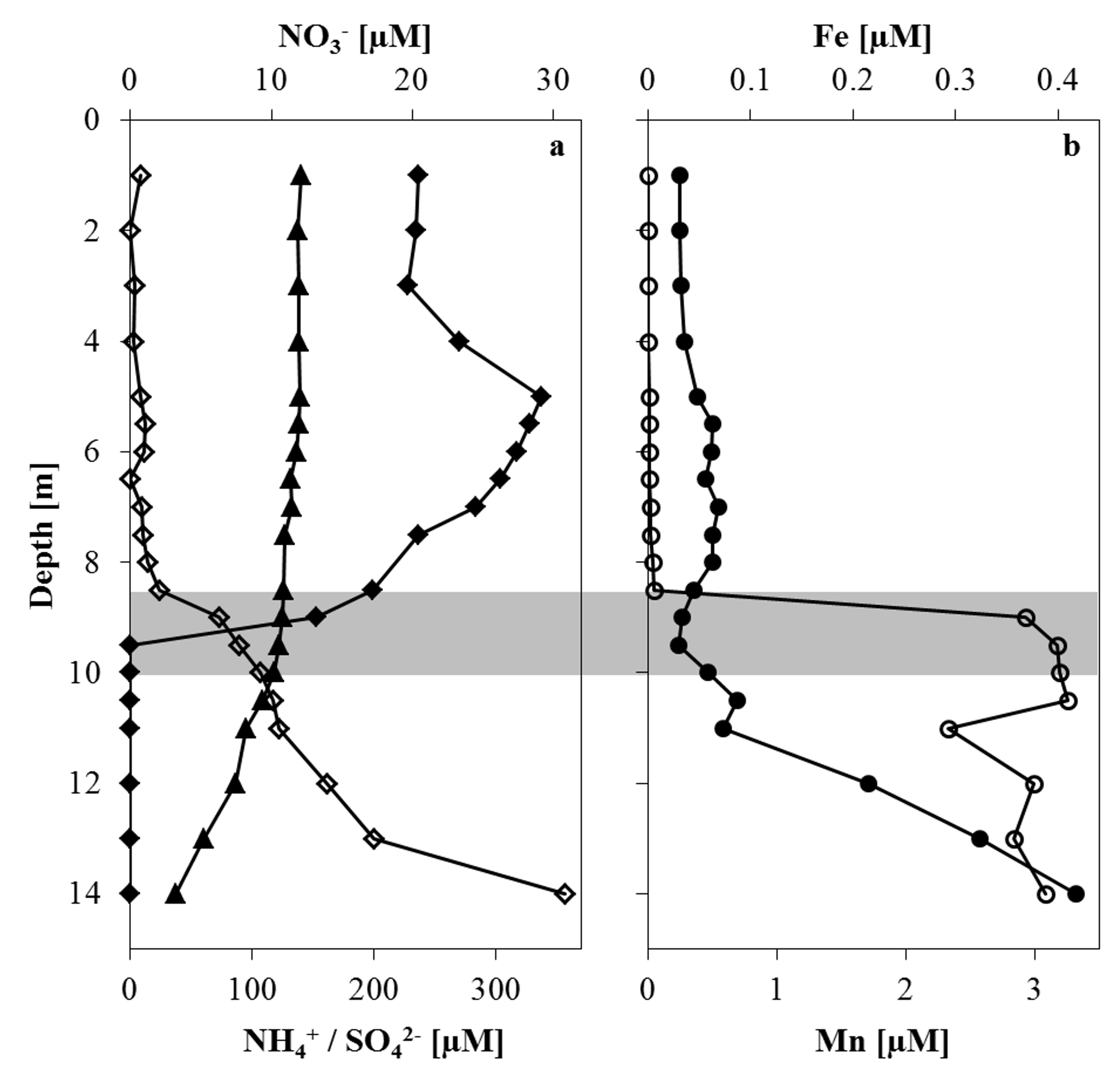

Supplement: S1 Fig — (a) Concentration depth profiles of nitrate (solid diamonds), ammonium (empty diamonds) and sulfate (solid triangles); and (b) dissolved iron (solid circles) and dissolved manganese (empty circles). The grey shading denotes the zone of methane oxidation. (TIF) [file pone.0132574.s001.tif]

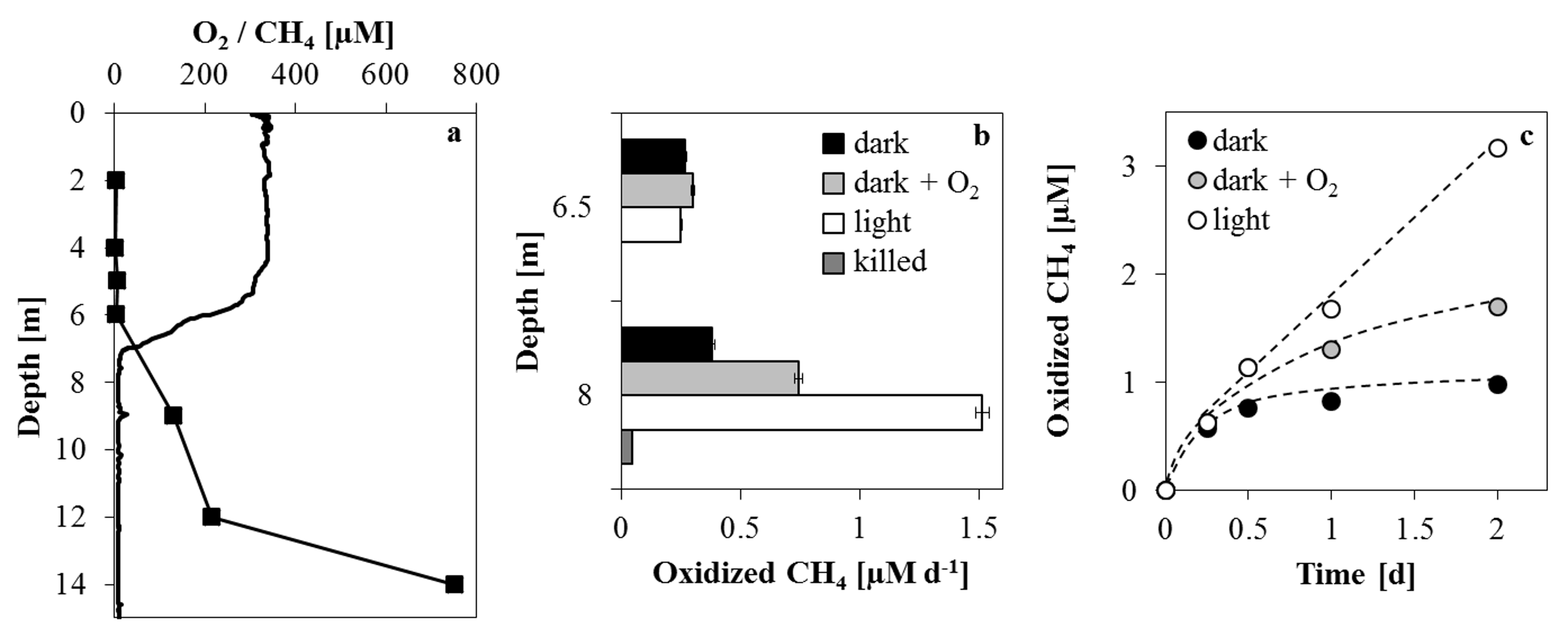

Supplement: S2 Fig — (a) Concentration depth profiles of oxygen (solid line) and methane (solid squares). (b) Methane oxidation rates from 6.5 and 8 m depth in dark conditions, with the addition of O2 (in the dark), in light conditions and the killed control. (c) Incubation time series from 8 m depth in the dark, with supplemented O2 (in the dark) and in the light. Bulk methane turnover (b) and oxidation time series (c) from 8 m depth were measured in the same incubation. (TIF) [file pone.0132574.s002.tif]

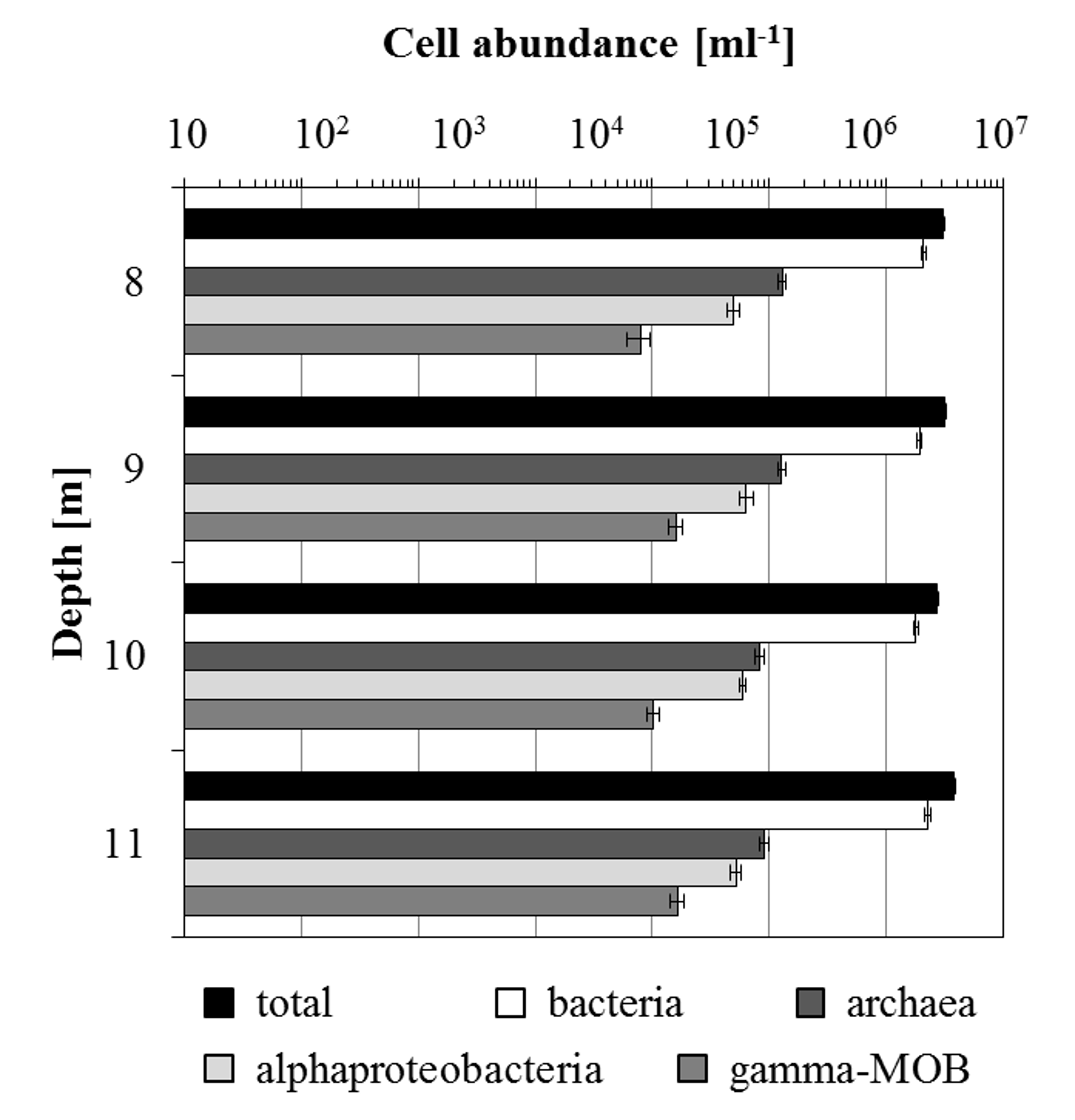

Supplement: S3 Fig — Total cell numbers of DAPI counted cells, bacteria (probe mix EUB338 I-III), archaea (probe ARCH915), alphaproteobacteria (probe alfa968) and gamma-MOB (probe mix Mgamma84+705) at incubation depths. Error bars denote the standard error of the mean of counted fields of view (20). Note the logarithmic scale. (TIF) [file pone.0132574.s003.tif]

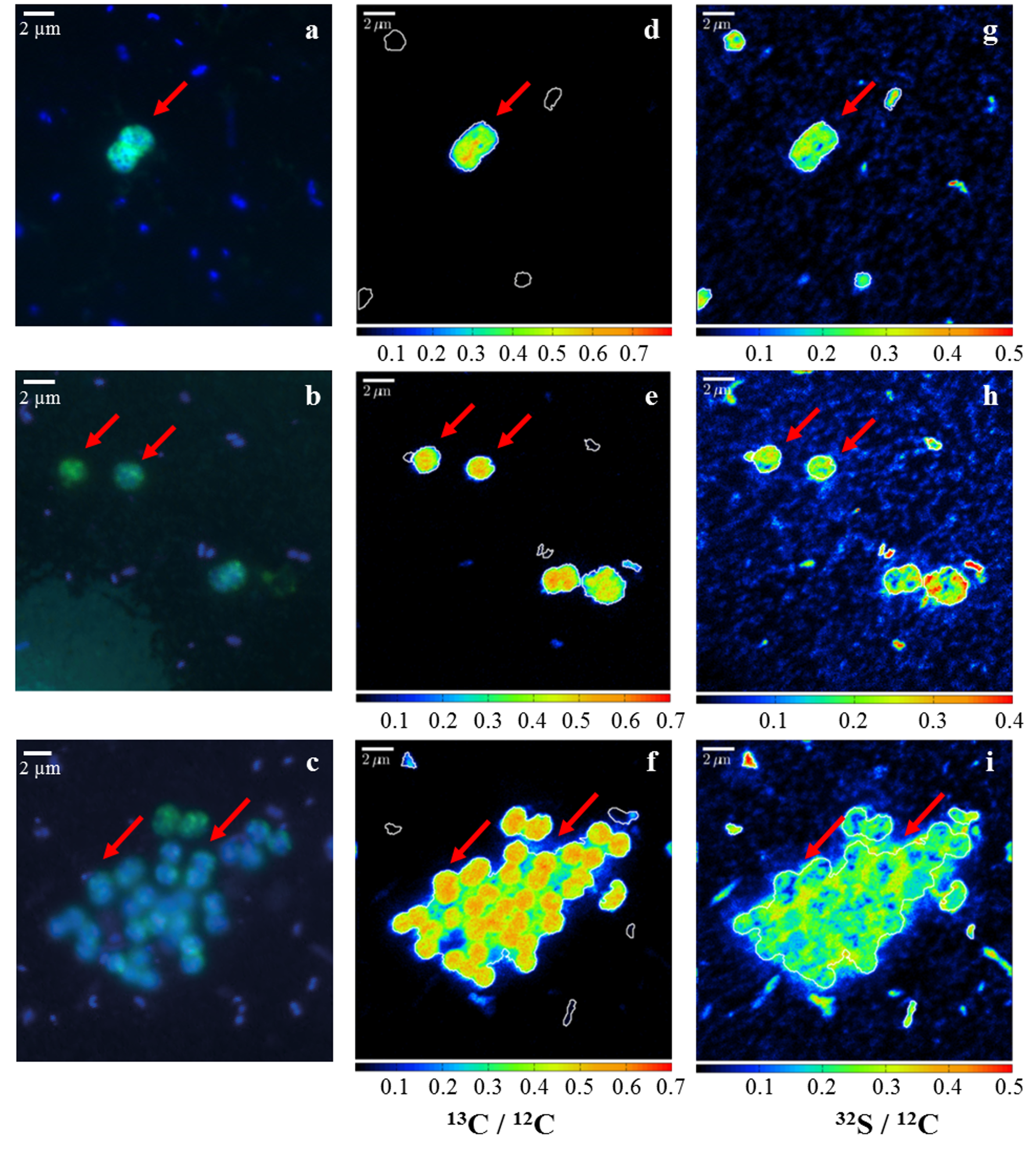

Supplement: S4 Fig — Fluorescent micrographs of gamma-MOB (a-c) visualized by DAPI (blue) and in situ hybridization with Mgamma 84+705 probes (green). Corresponding nanoSIMS images (d-f) of 13C/12C and (g-i) 32S/13C ratios after 2 d in light conditions (upper panel), after 2 d amended with oxygen (middle panel) and after 7 d in light conditions (lower panel). All scale bars are 2 μm and red arrows indicate hybridized/measured cells. (TIF) [file pone.0132574.s004.tif]

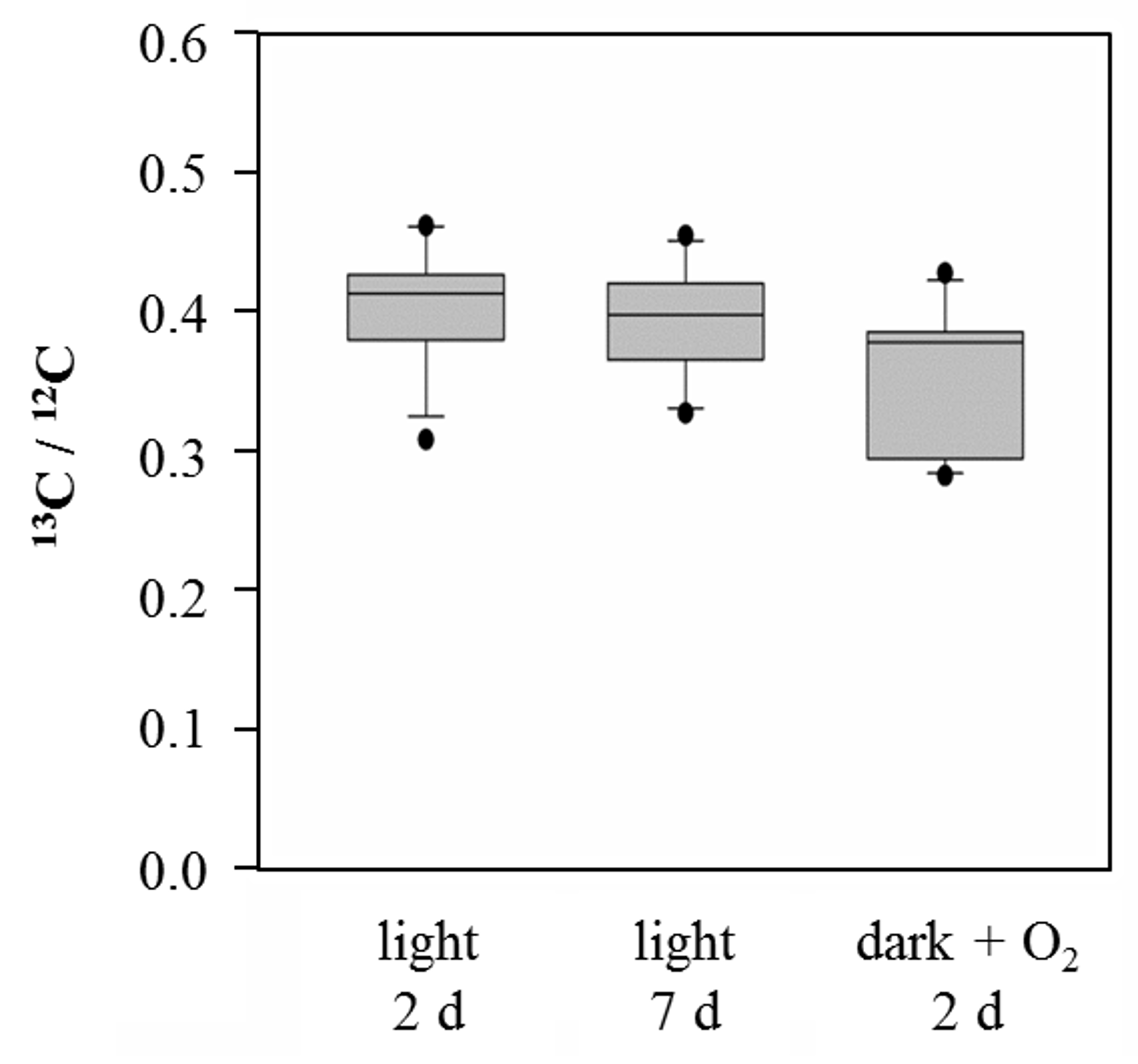

Supplement: S5 Fig — 13C-CH4 uptake (represented as 13C/12C ratios) by single gamma-MOB in the light incubation (after 2 d, n = 12 cells and 7 d, n = 10 cells) and with supplemented O2 (after 2 d, n = 11 cells). (TIF) [file pone.0132574.s005.tif]
