# Supplementary material for: Light-Dependent Aerobic Methane Oxidation Reduces Methane Emissions from Seasonally Stratified Lakes
Source: PLoS One. 2015 Jul 20;10(7):e0132574. doi: 10.1371/journal.pone.0132574 (PMC4508055; doi:10.1371/journal.pone.0132574)
Supplement: S1 Table — HRP probes along with their specificity, applied permeabilization, % [v/v] formamide in the hybridization buffer and reference. (PDF) [file pone.0132574.s006.pdf]

Probe	Specificity	Permeabili-zation	% Formamide	Probe sequence (5´-3´)	Reference
AAA-FW-641	AOM-associated archaea, freshwater cluster	Proteinase K	60	GGT CCC AAG CCT ACC AGT	[1]
AAA-FW-834				TGC GGT CGC ACC GCA CCT	
Alfa968	Alphaproteobacteria	Lysozyme	35	GGT AAG GTT CTG CGC GIT	[2]
ANME-1-350	ANME-1	Proteinase K	40	AGT TTT CGC GCC TGATGC	[3]
ANME-2-538	ANME-2	SDS	40	GGC TAC CAC TCG GGC CGC	[4]
ARCH915	Most archaea	Proteinase K	35	GTG CTC CCC CGC CAA TTC CT	[5]
EUB338 I-III	Most bacteria	Lysozyme	35	GCT GCC TCC CGT AGG AGT<br>GCA GCC ACC CGT AGG TGT<br>GCT GCC ACC CGT AGG TGT	[6]
Ma450	Alpha-MOB	SDS	20	ATC CAG GTA CCG TCA TTA TC	[7]
Mgamma84	Gamma-MOB	Lysozyme	20	CCA CTC GTC AGC GCC CGA	[7]
Mgamma705				CTG GTG TTC CTT CAG ATC	
NON338	Negative control	Lysozyme	35	ACT CCT ACG GGA GGC AGC	[8]

1. Schubert CJ, Vazquez F, Lösekann-Behrens T, Knittel K, Tonolla M, Boetius A (2011) Evidence for anaerobic oxidation of methane in sediments of a freshwater system (Lago di Cadagno). FEMS microbiology ecology 76: 26-38.
2. Neef A (1997) Anwendung der in situ-Einzell-Identifizierung von Bakterien zur Populations-Analyse in komplexen mikrobiellen Biozönosen. PhD thesis, Technische Universität München.
3. Boetius A, Ravenschlag K, Schubert CJ, Rickert D, Widdel F, Gieseke A, et al. (2000) A marine microbial consortium apparently mediating anaerobic oxidation of methane. Nature 407: 623-626.
4. Treude T, Niggemann J, Kallmeyer J, Wintersteller P, Schubert CJ, Boetius A, et al. (2005) Anaerobic oxidation of methane and sulfate reduction along the Chilean continental margin. Geochimica et Cosmochimica Acta 69: 2767-2779.
5. Stahl D, Amann R (1991) Development and application of nucleic acid probes in bacterial systematics; Goodfellow ESM, editor. Chichester, UK: John Wiley & Sons Ltd. 205–248 p.
6. Daims H, Brühl A, Amann R, Schleifer K-H, Wagner M (1999) The Domain-specific Probe EUB338 is Insufficient for the Detection of all Bacteria: Development and Evaluation of a more Comprehensive Probe Set. Systematic and Applied Microbiology 22: 434-444.
7. Eller G, Frenzel P (2001) Changes in activity and community structure of methane-oxidizing bacteria over the growth period of rice. Applied and environmental microbiology 67: 2395-2403.
8. Wallner G, Amann R, Beisker W (1993) Optimizing fluorescent in situ hybridization with rRNA-targeted oligonucleotide probes for flow cytometric identification of microorganisms. Cytometry 14: 136-143.
