# Supplementary material for: Light-Dependent Aerobic Methane Oxidation Reduces Methane Emissions from Seasonally Stratified Lakes
Source: PLoS One. 2015 Jul 20;10(7):e0132574. doi: 10.1371/journal.pone.0132574 (PMC4508055; doi:10.1371/journal.pone.0132574)
Supplement: S2 Table — Primary production rates were quantified at in situ conditions 2 d after all other physicochemical profiles were taken and methane oxidation rate experiments were set up. (PDF) [file pone.0132574.s007.pdf]

| **Depth [m]** | **Primary production [μM $d^{-1}$]** |
|---|---|
| 8 | 4.34 ± 0.35 |
| 9 | 2.71 ± 0.08 |
| 10 | 1.37 ± 0.14 |
| 11 | 0.43 ± 0.16 |
